# Modulation of Kombucha Functionality by Whey Protein-Encapsulated *Lactobacillus*: Effects on Bioactive Properties

**DOI:** 10.3390/foods15071258

**Published:** 2026-04-07

**Authors:** Tara Budimac, Aleksandra Ranitović, Olja Šovljanski, Jelena Vulić, Jasmina Vitas, Nevenka Gligorijević, Anja Vučetić, Ana Tomić, Radomir Malbaša, Dragoljub Cvetković

**Affiliations:** 1 Faculty of Technology Novi Sad, University of Novi Sad, 21000 Novi Sad, Serbia; tara.budimac@uns.ac.rs (T.B.); oljasovljanski@uns.ac.rs (O.Š.); jvulic@uns.ac.rs (J.V.); vitasj@uns.ac.rs (J.V.); anja.saveljic@uns.ac.rs (A.V.); anav@uns.ac.rs (A.T.); rmalbasa@uns.ac.rs (R.M.); cveled@uns.ac.rs (D.C.); 2 Department of Experimental Oncology, Institute for Oncology and Radiology of Serbia, Pasterova 14, 11000 Belgrade, Serbia; nevenka.gligorijevic@gmail.com

**Keywords:** enriched kombucha fermentation, lactic acid bacteria (LAB), whey protein encapsulation, probiotic viability, in vitro bioactivity, antimicrobial activity, antiproliferative activity, organic acids, fermentation microbiology

## Abstract

Kombucha is a fermented beverage produced using a symbiotic consortium of acetic acid bacteria and yeasts, often marketed for its health-promoting properties. However, probiotic bacteria in kombucha are typically present at inconsistent levels and may not remain viable during fermentation. In this study, three *Lactobacillus* strains (*Lacticaseibacillus rhamnosus* ATCC 53103 (*L. rhamnosus*), *Lactiplantibacillus plantarum* subsp. *plantarum* ATCC 14917 (*L. plantarum*) and *Lentilactobacillus hilgardii* (*L. hilgardii*) isolate) were encapsulated in whey protein using the lyophilization method and added individually at the start of kombucha fermentation. Lactic acid bacteria (LAB)–enriched kombucha samples were evaluated for chemical composition (polyphenols, flavonoids, vitamin C and organic acids) and functional properties (antimicrobial, antiproliferative, antioxidant and anti-inflammatory activities) and compared to a traditionally obtained control kombucha, primarily demonstrating in vitro and experimental assessment. Encapsulation maintained LAB viability above 6–7 log CFU/mL throughout fermentation, producing kombucha with enhanced microbial stability. LAB–enriched samples exhibited increased L-lactic acid and antimicrobial activity. *L. rhamnosus* and *L. hilgardii*–enriched samples exhibited increased antiproliferative and anti-inflammatory activities, which may be associated with strain-dependent production of organic acids, polyphenol modulation and LAB-derived bioactive metabolites. Antioxidant activity varied depending on assay, and *L. rhamnosus*–enriched kombucha showed higher anti-inflammatory activity. These findings demonstrate that whey protein encapsulation can preserve LAB during fermentation, enhance specific bioactive properties and provide a platform for developing functional kombucha beverages with potential applications in the food industry.

## 1. Introduction

Kombucha is a traditional fermented beverage that has gained popularity in recent decades. Although the knowledge about the origins of kombucha has been lost over the years, it is believed that fermented tea was first used in northeastern China (Manchuria region) in 220 BCE due to its detoxifying and energising properties. With the expansion of trade routes, kombucha was first introduced to Russia and then to Eastern Europe. Since then, its popularity has grown, and it has been presented on the market with various new flavours. Today, kombucha can be purchased in retail stores worldwide, as well as the tea fungus itself for home preparation [1].

This traditional beverage was originally obtained by fermenting sweetened black or green tea (*Camellia sinensis* L.), but other teas and alternative substrates can also be used for its preparation. Tea fermentation, which lasts from several days to two weeks, is the product of a symbiotic consortium of acetic acid bacteria (AAB) and osmophilic yeasts (SCOBY). In the symbiotic community, yeasts hydrolyse sucrose into glucose and fructose using the enzyme invertase and produce ethanol through glycolysis. Further, AAB uses glucose to produce gluconic acid and ethanol to produce acetic acid. They also produce cellulose, which appears as a pellicle on the surface of the fermentation liquid, and into which yeasts and AAB cells are incorporated. The introduction of LAB into this consortium may alter fermentation ecology through both competitive and cooperative interactions. LAB can compete with native microorganisms for available sugars, while simultaneously contributing to metabolite pools through the production of lactic acid and other bioactive compounds [2]. In addition, cross-feeding interactions and environmental factors such as pH and oxygen availability play a key role in shaping microbial succession and metabolic outputs. Therefore, incorporation of LAB into kombucha should be considered as a functional enrichment strategy, but also as a factor influencing the overall microbial balance and fermentation dynamics [1,3,4]. Kombucha consists of a wide range of chemical compounds such as polyphenols, organic acids, vitamins, minerals, carbohydrates, enzymes, etc., which are either products of the metabolism of SCOBY or originate from the substrate itself, with their structures potentially being modified and transformed into new compounds during fermentation [5]. Various bioactive properties have been attributed to kombucha, including antioxidant, antimicrobial, antiproliferative and detoxifying effects, largely based on in vitro and experimental data [6]. However, it is important to emphasise that evidence supporting these effects in humans remains limited. Therefore, these reported properties should be interpreted as indicative of potential rather than confirmed health benefits [1].

In some studies, lactic acid bacteria (LAB) are also present in kombucha beverages [7,8,9]. These bacteria produce lactic acid as the main or sole product of carbohydrate fermentation and consist of a heterogeneous group of phylogenetically related microorganisms. Among this group, *Lactobacillus* species produce organic substances that contribute to the taste, texture and aroma of the product, as well as prevent spoilage. In addition, they may have therapeutic and health benefits for consumers, which are associated with the probiotic properties of these bacteria [10]. Due to their presence, kombucha is often labelled as a probiotic product, even though the number of probiotic bacteria is often inconsistent [3].

Probiotics are defined as “*live microorganisms which, when administered in adequate amounts, confer a health benefit on the host*” [11]. Public awareness of the health benefits of fermented foods and beverages is increasing, and as a result, more functional foods containing probiotics are appearing on the market. To achieve potential health effects, probiotic products must contain a recommended minimum of 6–7 log CFU/mL or g of probiotic bacteria at the time of consumption [12]. However, probiotic bacteria usually do not remain viable in sufficient concentrations under often unfavourable conditions encountered during fermentation and storage, including low pH, temperature fluctuations and oxygen exposure [13].

One of the main technologies used to protect and enhance the survival and viability of probiotic bacteria, thus ensuring product stability and targeted release in the intestines in adequate amounts, is the encapsulation method. During the encapsulation process, small quantities of the core material containing nutritional or therapeutic substances and probiotic bacteria are enclosed within a carrier wall to form microcapsules [14].

Various functional activities and the chemical composition of kombucha have been extensively investigated, including antimicrobial [15,16], antioxidant [17,18], anti-inflammatory [19] and antiproliferative activities [20,21]. In addition, several studies have already examined the effects of LAB addition to kombucha, reporting changes in the chemical compound profile (e.g., lactic and glucuronic acid production [22,23] and flavour profile [24]), as well as antibacterial and antioxidant activities [25]. Also, the antidiabetic, antihypertensive and antihypercholesterolemic effects of kombucha supplemented with encapsulated *L. rhamnosus* have been investigated [3], demonstrating more pronounced activity compared to control kombucha. However, there is a lack of studies evaluating the effect of whey protein-based encapsulation on LAB viability during kombucha fermentation, together with the impact of the resulting encapsulates on the chemical composition and functional properties of kombucha.

Therefore, this study hypothesised that encapsulation of LAB in a whey protein matrix would enhance their stability during fermentation and influence the bioactive profile of kombucha in a strain-dependent manner. Therefore, this study aimed to investigate the effect of whey protein–encapsulated LAB on (i) cell viability during fermentation and (ii) selected in vitro bioactive properties of kombucha, including antimicrobial, antioxidant, antiproliferative and anti-inflammatory activities. In this study, probiotic kombucha beverages were produced by the addition of two referenced LAB strains (*Lacticaseibacillus rhamnosus* ATCC 53103 (*L. rhamnosus*), *Lactiplantibacillus plantarum* subsp. *plantarum* ATCC 14917 (*L. plantarum*)) and one isolated strain (*Lentilactobacillus hilgardii* (*L. hilgardii*)). All strains were individually encapsulated in whey protein using the lyophilisation method and added at the beginning of kombucha fermentation. The LAB–enriched samples were then evaluated for their chemical composition, including organic acids, vitamin C, total phenols and flavonoids, as well as for their functional properties such as antimicrobial, antioxidant, antiproliferative and anti-inflammatory activities. All results were compared to a control sample of traditionally produced kombucha without the addition of encapsulated probiotics.

## 2. Materials and Methods

### 2.1. Preparation of Probiotic Cultures

Referenced probiotic bacterial strains *L. rhamnosus* ATCC 53103, *L. plantarum* ATCC 14917 and *L. hilgardii* (previously isolated from sourdough [26]) were stored at −80 °C in an ultra-low-temperature deep freezer (Snijders Labs, Tilburg, Norway) in *Lactobacillus* MRS Broth (Himedia, Mumbai, India), supplemented with 200 g/L of glycerol, at the Laboratory of Microbiology, Faculty of Technology, University of Novi Sad, Novi Sad, Serbia. The strains were recultured by evenly spreading 25 µL of the strain mixture on the surface of several previously poured *Lactobacillus* MRS Agar (Himedia, Mumbai, India) plates, before use. The plates were stored anaerobically in Anaerocult A^®^ (Merck, Darmstadt, Germany) at 30 °C for 48–72 h. After cultivation, a suspension in saline peptone solution (0.85% NaCl, 0.1% peptone) was made. The concentrations of LAB were estimated at 3 × 10^9^ CFU/mL by comparing with McFarland Standards [27]. McFarland was used only for inoculum standardisation, whereas LAB counts during fermentation were determined by plate enumeration as described below.

### 2.2. Encapsulation of LAB Cells

Encapsulation was performed according to Budimac et al. [3] with whey protein (BioTech USA, Szada, Hungary) used as carrier material.

### 2.3. Kombucha Preparation and Fermentation

Preparation and fermentation of kombucha were conducted as previously described by Budimac et al. [3]. Sampling was performed after 1 h of the addition of LAB encapsulates and for the next 4 days every day for microbiological (number of yeasts, AAB and LAB) and chemical (pH and titratable acidity (TA)) analysis. A fermentation period of 4 days was selected to capture early stage microbial dynamics and to preserve LAB viability under conditions that are less inhibitory than those observed during prolonged fermentation. Microbiological (number of yeasts and AAB) and chemical analyses (pH and TA) were performed using methods previously described by Ranitovic et al. [28]. The number of LAB was determined as described by Budimac et al. [3].

### 2.4. Analysis of Organic Acids

Reversed-phase chromatography was used for measuring acetic, succinic, oxalic, tartaric, formic, malic, malonic and citric acid content on Agilent 1100 Series HPLC, Arcade, NY, USA, according to Vukmanović et al. [29]. Calibration curve equations for determined organic acids are as follows: y = 702.63x + 6.8199 (acetic), y = 759.29x + 0.2236 (malonic), y = 749.53x + 4.1471 (formic) and y = 5052.1x + 137.4 (oxalic); R 2 values amounted 0.9999 and higher; y represents the area of the chromatographic peak and x the content of the organic acid in mg/mL. Results were expressed in milligrams of organic acid per millilitre of the sample

D-lactic acid and L-lactic acid content were determined by using the D-/L-lactic acid kit (Megazyme, Co., Wicklow, Ireland, K-DLATE 06/08), according to the manufacturer’s instructions. Results were expressed in grams of organic acid per litre of the sample.

### 2.5. Analysis of Vitamin C

Vitamin C was determined as previously described by Vitas et al. [30], and the results are expressed as milligrams per litre of the sample.

### 2.6. Total Polyphenol and Flavonoid Content and Antioxidant Capacity

The total polyphenolic contents for the kombucha samples were determined spectrophotometrically using the microscale-adapted Folin–Ciocalteau method. The absorbance was measured at 750 nm using distilled water as a blank. The obtained results were expressed as gallic acid equivalents (GAE) per mL sample, based on a calibration curve prepared with gallic acid.

The aluminium chloride colourimetric assay was adapted for a 96-well microplate and used to spectrophotometrically determine the content of the flavonoids found in kombucha samples. The absorbance of the prepared reaction mixture was measured at 510 nm. The results were expressed as rutin equivalents (RE) per mL sample, based on a calibration curve prepared with rutin.

Three antioxidant tests were conducted: 2,2-diphenyl-1-picrylhydrazyl (DPPH), 2,2′-azino-bis-3-ethylbenzothiazoline-6-sulphonic acid (ABTS) and reducing power (RP), which are based on radical scavenging ability and reducing capacity of the samples. Trolox was used as a standard antioxidant, and the results were expressed as mmol Trolox equivalents (TE) per 100 mL of sample (mmol TE/100 mL), based on calibration curves.

All methods were previously described in detail by Ranitovic et al. [28].

### 2.7. Analysis of Polyphenolic Compounds

Kombucha samples were subjected to HPLC analysis coupled with a DAD detector (Shimadzu Prominence, Kyoto, Japan), to determine and measure the polyphenolic constituents as described by Aćimović et al. [31]. The results are expressed as micrograms per millilitre of sample.

### 2.8. Antimicrobial Activity

Antimicrobial assessment was conducted using the disk-diffusion method described in the work of Vukmanović et al. [29]. As test microorganisms, the following strains were used: Gram-negative bacteria (*Escherichia coli* ATCC 25922, *Salmonella* Typhimurium ATCC 14028, *Pseudomonas aeruginosa* ATCC 27853), Gram-positive bacteria (*Listeria monocytogenes* ATCC 35152, *Bacillus cereus* ATCC 11778, *Staphylococcus aureus* ATCC 25923), yeast (*Candida albicans* ATCC 10231) and moulds (*Aspergillus brasiliensis* ATCC 16404, *Penicillium aurantiogriseum* ATCC 16025). Samples for the determination of the antimicrobial activity included: LAB–enriched kombucha samples after 4 days of fermentation, as well as the control samples; uninoculated fermentation media (black tea 3 g/L); acetic acid solutions of appropriate concentrations as in kombucha beverages: 3.54 g/L (*L. plantarum*), 4.85 g/L (*L. hilgardii*), 6.09 g/L (*L. rhamnosus*) and 5.14 g/L (Control); kombucha neutralized to pH 7 with 0.1 mol/L NaOH and kombucha heated to boiling for 10 min to denature proteins. All samples were filtered through a sterile microfilter (0.22 μm) to remove cells before testing. The evaluation of antimicrobial activity was carried out in triplicate, and results are presented as the diameter of the halo zone (mm) ± standard deviation.

### 2.9. Anti-Inflammatory Activity

In vitro evaluation of the anti-inflammatory characteristics was conducted with the test protein denaturation according to Ranitovic et al. [28]. The results were expressed as percentage inhibition of protein denaturation.

### 2.10. Antiproliferative Activity

Human colon adenocarcinoma HT-29, human breast adenocarcinoma MCF-7 and human cervical adenocarcinoma HeLa cells were maintained as a monolayer culture in Roswell Park Memorial Institute (RPMI) 1640 nutrient medium (Sigma Chemicals Co., St. Louis, MO, USA). RPMI 1640 nutrient medium was prepared in sterile deionised water, supplemented with penicillin (192 IU/mL), streptomycin (200 µg/mL), 4-(2-hydroxyethyl) piperazine-1-ethanesulfonic acid (HEPES) (25 mM), L-glutamine (3 mM) and 10% of heat-inactivated fetal calf serum (FCS) (pH 7.2). The cells were grown at 37 °C in 5% CO_2_ and a humidified air atmosphere. The antiproliferative activity of kombucha samples was tested using the 3-(4,5-dymethylthiazolyl)-2,5-diphenyltetrazolium bromide (MTT) assay [32]. Briefly, HT-29 (3000 cells/well), MCF-7 (3000 cells/well) and HeLa (2000 cells/well) cells were seeded in 96-well plates (SPL Life Sciences Co., Ltd., Pocheon-si, Korea) and incubated for 24 h, to allow cells to attach and enter into the exponential growth phase before the addition of examined samples. Cells were treated with samples derived from investigated LAB–enriched kombucha samples (*L. plantarum*, *L. hilgardii*, *L. rhamnosus*) and a control kombucha sample (Control) in desired concentrations. All samples were filtered through 0.22 µm pore-size filters (Millipore, Burlington, MA, USA) before use. All experimental treatments were evaluated compared with untreated controls (i.e., controls treated with medium only). After 48 h of incubation, 20 μL MTT solution (5 mg/mL in PBS, pH 7.2) was added, followed by 4 h of incubation at 37 °C. Formazan crystals were dissolved using 10% SDS, and the absorbance was measured at 570 nm after 24 h, on an enzyme-linked immunosorbent assay reader (MULTISCAN SkyHigh, Thermo Scientific, Waltham, MA, USA). Results were displayed as cell survival percentages.

### 2.11. Statistical Analysis

All tested parameters were determined in triplicate. The obtained values are presented as a mean ± standard deviation. Analysis of variance (ANOVA) and Tukey’s HSD test for comparison of sample means were used to analyse variations in the observed parameters among the samples. The data were processed statistically using the software package OriginPro 2026 (OriginLab Corporation, Northampton, MA, USA).

## 3. Results and Discussion

### 3.1. Kombucha Fermentation Parameters

The whey protein-encapsulated *L. rhamnosus*–enriched kombucha sample was previously investigated for its fermentation parameters, and these results are reported in a study by Budimac et al. [3]. Previously reported fermentation parameters for *L. rhamnosus*–enriched kombucha are briefly summarised here to facilitate comparison with newly obtained data on bioactive properties. The sample showed the highest final TA (6.09 ± 0.03), and the concentration of acetic acid bacteria (AAB) increased from 6.88 to 7.58 log CFU/mL on day 1, remaining mainly stable and reaching a maximum of 7.83 log CFU/mL on day 4. Yeast numbers slightly declined by day 2 (6.11 ± 0.16) and then remained relatively stable until the end of fermentation. LAB growth was also highest in this sample, reaching 7.71 ± 0.03 log CFU/mL on day 4. While these fermentation parameters have been reported previously, the chemical composition and bioactive properties of this LAB–enriched kombucha have not yet been examined, which is the aim of the present study.

**Figure 1:**
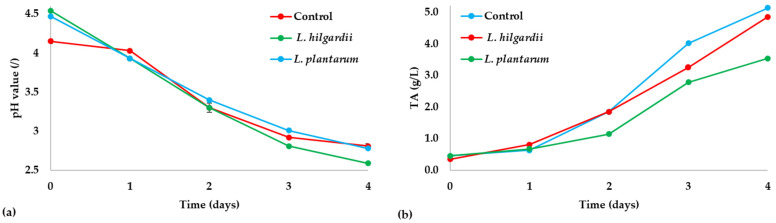
Kombucha fermentation parameters: (**a**) pH value and (**b**) titratable acidity (TA). Values are expressed as mean ± SD (*n* = 3).

The initial pH of uninoculated black tea was 7.30 and decreased significantly after inoculation, ranging from 4.15 ± 0.02 for the control sample to 4.47 ± 0.03 for the *L. plantarum*- and 4.54 ± 0.01 for the *L. hilgardii*–enriched sample (Figure 1a). Over four days of fermentation, all the samples showed a decrease in pH values. On the other hand, TA increased gradually in all samples, with the *L. rhamnosus*–enriched sample reaching the highest value of 6.09 ± 0.03 [3], followed by the control and *L. hilgardii*–enriched sample (5.14 ± 0.03 and 4.85 ± 0.05, respectively), while *L. plantarum*–enriched sample showed the lowest value of 3.54 ± 0.00 g/L on day 4 (Figure 1b). Although formal kinetic modelling was not performed, the observed microbial trends indicate an initial adaptation phase followed by stabilisation of LAB populations and a gradual increase in AAB counts. This pattern suggests active microbial interactions during early fermentation, with LAB maintaining stable populations while AAB proliferation continues throughout the fermentation period.

These results are in accordance with the standard growth curve during kombucha fermentation as shown in other studies [3,24,26,33,34]. Acetic acid represents the predominant organic acid in the fermentation medium [35]. Besides acetic acid, the metabolic activity of SCOBY microorganisms results in the production of various other organic acids, including lactic acid formed by LAB in enriched kombucha samples [3]. Accumulation of these acids over the course of fermentation causes a linear increase in TA, which consequently leads to a gradual decrease in pH values that eventually stabilise with extended fermentation time [35].

**Figure 2:**
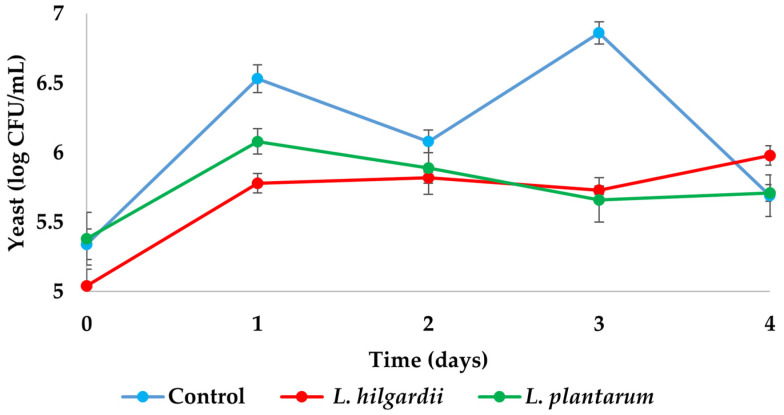
Number of yeasts (log CFU/mL) during kombucha fermentation. Values are expressed as mean ± SD (*n* = 3).

Control samples maintained higher counts of yeast during fermentation than LAB–enriched samples in the first 3 days (6.86 ± 0.08 log CFU/mL), after which a slight decline in all samples was observed (Figure 2). As no significant differences in yeast counts were observed between the LAB–enriched kombucha samples and the control on day 4, it can be concluded that the addition of whey protein–LAB encapsulates did not affect yeast populations during fermentation. A similar pattern in yeast numbers was observed by Cvetkovic et al. [26], where there was an increase in the number of yeasts on the first day, after which the number was relatively stable, with small fluctuations until the end of kombucha fermentation.

**Figure 3:**
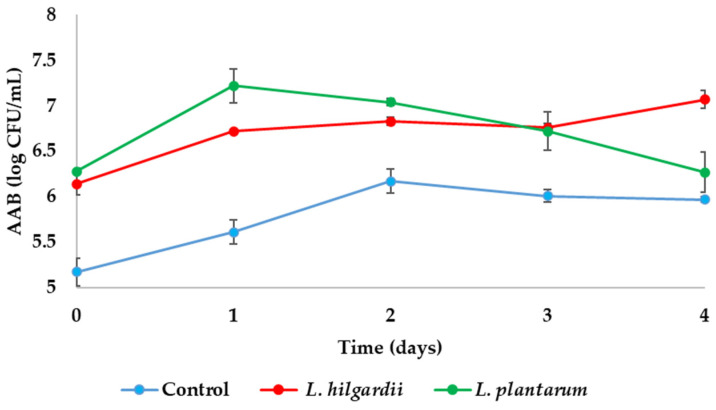
Number of acetic acid bacteria (AAB) during kombucha fermentation. Values are expressed as mean ± SD (*n* = 3).

In contrast, some differences were noticed in AAB numbers among the tested samples (Figure 3). In general, higher numbers of AAB were noticed in LAB–enriched samples rather than the control. *L. rhamnosus*–enriched sample exhibited the highest number on day 4 (7.83 ± 0.02 log CFU/mL) in a previous study [3], followed by *L. hilgardii*–(7.07 ± 0.1 log CFU/mL) and *L. plantarum*–enriched sample (6.27 ± 0.22 log CFU/mL), while control remained at 5.97 ± 0.03 log CFU/mL in the present study. In a study by Yang et al. [23], it was reported that the presence of LAB during fermentation can promote the growth and proliferation of other microorganisms, including AAB, as well as the production of acetic acid and other metabolites, which may explain the higher AAB counts observed in LAB–enriched kombucha samples compared to the control in the present study. Similar results were reported by Majid et al. [22] and Cvetković et al. [26], who observed higher AAB concentrations at the end of fermentation in samples supplemented with free LAB cells than in control samples. The observed increase in AAB populations in LAB–enriched samples may be explained by metabolic interactions within the fermentation system. LAB contribute to the production of organic acids and intermediate metabolites that can influence the growth environment and microbial balance. In addition, LAB activity may indirectly support AAB proliferation by modifying substrate availability and creating favourable ecological conditions, such as gradual acidification and metabolic cross-feeding. These interactions highlight the complexity of microbial dynamics in kombucha fermentation and suggest that LAB incorporation can influence not only product composition but also microbial ecosystem behaviour.

**Figure 4:**
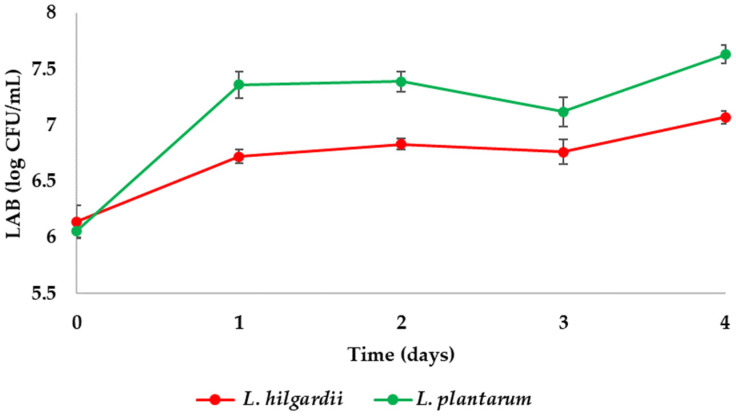
Number of lactic acid bacteria (LAB) during kombucha fermentation (Control did not contain LAB number (not detected); therefore, control data is excluded from the graph for better visual presentation of the obtained results of other samples). Values are expressed as mean ± SD (*n* = 3).

Changes in the number of LAB during fermentation are shown in Figure 4. LAB showed a slight growth in numbers in the first 24 h after the addition, after which the numbers remained relatively stable until the end of fermentation and showed no significant differences between the enriched samples, with *L. rhamnosus*–enriched sample having the highest number of 7.71 ± 0.03 log CFU/mL [3], followed by *L. plantarum*- with 7.63 ± 0.08 log CFU/mL and *L. hilgardii*–enriched sample with 7.55 ± 0.06 log CFU/mL, while LAB were absent in control sample (Figure 4). The belonging bacteria of the *Lactobacillus* genus can grow in a pH varying from 4.5 to 6.5, with optimal pH being 5.5–6.2, while some strains could grow in lower pH [36]. Given that the pH often decreases below 3 during kombucha fermentation, it could be concluded that free *Lactobacillus* cells would not remain viable in these conditions. Limited research has successfully demonstrated the production of kombucha beverages that fulfil the criteria of probiotic products, primarily due to the decline in LAB populations during fermentation and storage.

In a previous study by Cvetković et al. [37], the same *L. rhamnosus* and *L. plantarum* strains used in the present study were introduced as free cells at the onset of a 5-day kombucha fermentation. The populations of both probiotic strains decreased drastically by the end of fermentation, reaching levels below 2.0 log CFU/mL on day 5, thereby supporting this observation. The same isolated *L. hilgardii* strain was used in a study by Cvetkovic et al. [26], where the numbers showed a decrease during the storage of kombucha. Other studies also report low survival rates of LAB strains during fermentation and storage of kombucha [38,39]. In contrast, Majid et al. [22] successfully produced a probiotic blue pea tea kombucha by inoculating *L. plantarum* Dad-13 at the beginning of fermentation, achieving a LAB concentration of 6.26 log CFU/mL after fermentation and 28 days of storage. These differing outcomes may be attributed to variations in LAB strains used, as well as differences in tea substrates employed for kombucha production. Previous studies showed good survival of *L. rhamnosus* encapsulated in pea and whey proteins [3], while in the current study, it is confirmed that encapsulation is an adequate method for preserving the number of LAB during fermentation, also including other strains such as *L. plantarum* and *L. hilgardii*.

### 3.2. Organic Acids

In kombucha, organic acids are mostly formed during fermentation by the SCOBY metabolic activity [40]. Organic acids are responsible for the sour taste and aroma of kombucha, and they participate in antimicrobial activity, which protects against contamination and extends the shelf life of kombucha [41]. AAB from kombucha produces acetic acid as one of the main metabolites when sucrose is used as a carbon source. It is the predominant organic acid found in kombucha, and its content generally increases continuously during the cultivation of SCOBY [42,43]. Acetic acid concentration in the present study ranged from 1.1689 to 2.1550 mg/mL (Table 1) after four days of fermentation and was the most dominant organic acid for all the samples except for *L. rhamnosus*–enriched kombucha, where L-lactic acid was present in higher amounts (Table 2). Cardoso et al. [44] also found that acetic acid was the most dominant acid in green and black tea kombucha, amounting to about 3 mg/mL after 10 days of fermentation, while Jayabalan et al. [43] found acetic acid was present in an amount of 2.44 mg/mL after 9 days. In addition to antimicrobial activity, acetic acid could contribute to lowering cholesterol and triglyceride levels, controlling blood sugar levels, helping prevent constipation and even increasing the body’s absorption of vital minerals from food [45]. Malonic, formic and oxalic acids were present in lower quantities and ranged from 0.1202 mg/mL for the *L. hilgardii*–enriched sample to 0.4190 mg/mL for the control, 0.0742 mg/mL for the *L. hilgardii*–enriched sample to 0.2303 mg/mL for the control and from 0.2169 mg/mL for *L. plantarum*–enriched sample to 0.6011 mg/mL for *L. rhamnosus*–enriched sample, respectively. A similar amount of oxalic acid was found after 10 days of kombucha fermentation, 0.43 mg/mL in a study by Neffe-Skonciska et al. [46]. On the other hand, oxalic acid was present in an amount of 2.12 mg/mL in a study by Vukmanovic et al. [41]. The higher amount than in the present study could be explained by different substrates, since winery effluent was used in the mentioned study, while malonic and formic acid contents were similar. Other organic acids, such as succinic, tartaric, malic and citric could also be found in kombucha as shown in several studies [5,41,46,47], but were not detected in the present study.

The concentration of D-lactic acid in LAB–enriched kombucha samples ranged from 0.12 ± 0.00 to 0.98 ± 0.00 g/L (Table 2). The highest D-lactic acid content was observed in the *L. plantarum*–enriched kombucha, followed by the *L. hilgardii*–enriched sample. In contrast, *L. rhamnosus*–enriched kombucha exhibited significantly lower levels, while the control sample contained only trace amounts of D-lactic acid (0.02 ± 0.00 g/L). The concentration of L-lactic acid varied significantly among LAB–enriched kombucha samples, ranging from 0.64 ± 0.01 to 5.22 ± 0.09 g/L (Table 2). Kombucha enriched with *L. rhamnosus* exhibited markedly higher L-lactic acid content compared to all other samples (*p* < 0.05). As previously mentioned, this sample also exhibited the highest TA [3], which is in accordance with high lactic acid content. *L. plantarum*– and *L. hilgardii*–enriched samples showed moderate but significantly different levels of L-lactic acid, while the control sample contained only trace amounts. Tukey’s HSD test confirmed significant differences among all enriched samples and the control.

Although lactic acid is not a characteristic compound of traditionally prepared kombucha, its presence has been reported in several studies [48]. Regular consumption of lactic acid has been associated with potential beneficial effects on certain physiological functions, including improved digestibility, stimulation of intestinal peristalsis, improved blood circulation, maintenance and balance of body pH, improved absorption of nutrients, etc. In addition, reduced levels of L-lactic acid in connective tissue have been reported in cancer patients, and kombucha containing this acid could potentially contribute as a dietary source [49]. Jayabalan et al. [43] examined a kombucha beverage prepared from green tea and concluded that it contained a higher concentration of lactic acid than kombucha prepared from black tea and tea waste material. The maximum value of 0.54 g/L total lactic acid was obtained on the 3rd day of fermentation. Malbaša et al. [50] also measured the lactic acid content after fermentation of kombucha on molasses and found that the tested acid was present in quantities reaching about 0.4 g/L, which is lower than in the present study, most likely due to the absence of LAB.

In the present study, *L. rhamnosus*–enriched kombucha exhibited a markedly higher L/D lactic acid ratio compared to other samples, primarily due to its substantially elevated L-lactic acid production and relatively low D-lactic acid levels. In contrast, *L. plantarum*- and *L. hilgardii*–enriched kombucha samples showed lower L/D ratios, reflecting a more balanced or D-lactic acid–biased production pattern. During fermentation, two types of lactate dehydrogenase (LDH) enzymes within LAB (L-lactate dehydrogenase (L-LDH) and D-lactate dehydrogenase (D-LDH)) produce the two chiral forms of lactic acid: L-lactic acid and D-lactic acid. Some LAB contain only a single type of LDH and thus produce only one chiral form of lactic acid. The predominance of L-lactic acid in the *L. rhamnosus*–enriched kombucha can be attributed to the strain’s stereospecific lactate dehydrogenase activity, favouring the formation of the physiologically preferred L-isomer [51]. Zhou and Hua [51] also reported that *L. rhamnosus* produces L-lactic acid due to the presence of L-LDH. It can be concluded that the predominance of L-lactic acid in the *L. rhamnosus*–enriched sample may be associated with strain-specific metabolic characteristics reported in the literature, particularly the activity of stereospecific LDH. However, enzyme activity was not directly measured in the present study.

This characteristic is particularly relevant from a food safety perspective, as excessive intake of D-lactic acid has been associated with adverse metabolic effects, especially in sensitive populations [52,53]. However, based on the recommendations of the World Health Organisation, the maximum allowed daily intake of D-lactic acid is 100 mg/kg of body weight [54]. For an adult with a body weight of 75 kg, this corresponds to an intake of 7500 mg/day. Bearing in mind that the highest recorded content of D-lactic acid was 985 mg/L in *L. plantarum*–enriched kombucha, and that the recommended daily amount of kombucha is up to two glasses (0.5 L), the maximum daily intake of D-lactic acid would be approximately 493 mg. This corresponds to ~6.6% of the maximum allowed daily intake, indicating that consumption of kombucha with added encapsulated LAB in this study does not pose a risk in terms of D-lactic acid intake. The control sample exhibited negligible concentrations of both L- and D-lactic acid, which further confirms that lactic acid production in enriched samples was primarily driven by the metabolic activity of the encapsulated LAB rather than by the native kombucha microbiota. Similar results in terms of L/D-lactic acid ratios were observed in a study by Cvetkovic et al. [26], where the content of D-lactic acid in the kombucha fermentation broth after the addition of several free LAB strains was higher than that of L-lactic acid for all wild strains except LAB strain isolated from 2 month old cheese, while the highest lactic acid concentrations (0.248 g/L D-lactic acid and 0.095 g/L L-lactic acid) were observed, which are lower than those reported in the present study. Majid et al. [22] added free *L. plantarum* cells to kombucha fermentation, and the total lactic acid content equated to 1.86 g/L, also lower than in the present study. The higher lactic acid concentrations observed in the present study compared to previous reports may be attributed to the use of encapsulated LAB rather than free cells. Encapsulation in whey protein matrices likely enhanced metabolic activity by protecting cells from acidic stress, ethanol and inhibitory organic acids inherent to kombucha fermentation [55].

### 3.3. Vitamin C

Vitamin C in kombucha is produced by the metabolic activity of certain strains of AAB, and the synthesised amount usually depends on the carbon source in the cultivation substrate, whereby sucrose stimulates the synthesis of vitamin C more than glucose and fructose [56]. Vitamin C is derived from glucose metabolism, especially by strains of the genus *Gluconobacter*. In addition, kombucha fermented with green or black tea contains high levels of vitamin C or ascorbic acid, given that these are also present in the tea leaves themselves [35]. Vitamin C belongs to antioxidants and has numerous desirable effects on human health, such as strengthening the immune system, collagen production, wound healing, teeth and gum health, regeneration of skin, cartilage, tendons, connective tissue, blood vessels, etc. [57,58]. Vitamin C content in LAB–enriched samples was similar and ranged from 2.24 mg/L in the *L. hilgardii* sample to 2.92 mg/L in the *L. rhamnosus* sample, while the control had a significantly higher level of vitamin C (4.54 mg/L) (Table 3). Vitamin C content in LAB–enriched kombucha samples was lower than in the control and generally lower than reported in previous studies [17,59], indicating that kombucha in this study contributes only modestly to dietary vitamin C intake [60]. This reduction may be partly explained by the metabolic activity of *Lactobacillus* strains, which can utilise ascorbic acid [61], converting it into organic acids, as well as by the inherent instability of vitamin C under fermentation conditions, especially in aqueous environments [62], and the presence of encapsulated LAB in whey protein may have further influenced vitamin C degradation. Overall, vitamin C levels may vary widely across kombucha studies due to differences in microbial communities, fermentation conditions, substrates used and methods of detection [6]. On the other hand, the lower vitamin C content observed in LAB–enriched samples may be associated with several factors, including potential microbial utilisation, chemical instability of ascorbic acid during fermentation and interactions within the fermentation matrix.

### 3.4. Total Polyphenol and Flavonoid Content and Antioxidant Capacity

Polyphenols in kombucha are produced from the tea leaves that are used [47]. The total content of phenolic compounds increases during kombucha fermentation, as complex molecules are degraded to smaller molecules by enzymes produced by SCOBY during this process [28,56]. The concentration of these compounds depends on the starter culture used, the length of fermentation and other fermentation conditions [63]. Table 4 shows the results of the investigated polyphenolic and flavonoid contents of LAB–enriched kombucha and control samples. Total polyphenol content varied from 0.26 to 0.39 mg GAE/mL. The highest value of total polyphenols was recorded in the *L. rhamnosus*-enriched sample (0.39 ± 0.05 mg GAE/mL), which was significantly different from the *L. plantarum*– and *L. hilgardii*–enriched samples (*p* < 0.05). The control sample showed moderately high polyphenolic content and did not show a statistically significant difference compared to the *L. rhamnosus* sample. On the other hand, total flavonoid content was lower and relatively uniform among all samples, ranging from 0.01 to 0.02 mg RE/mL, with no statistically significant differences (*p* > 0.05). These results indicate that the applied bacterial strains did not have a significant effect on the content of flavonoids, in contrast to the total polyphenolic compounds, where the strain-specific effect was more pronounced.

Polyphenols present in tea and therefore also in kombucha have pronounced antioxidant activity, the ability to “capture” free radicals, which has a favourable effect on the prevention of certain cancers, strengthens immunity, reduces inflammatory processes, etc. [35]. Antioxidant capacity was analysed using three antioxidant assays (Table 5). The results of the tested kombucha samples showed different patterns depending on the method used. The DPPH test showed statistically significant differences between the samples, with LAB–enriched samples having slightly lower activity compared to the control. On the other hand, the ABTS test did not show statistically significant differences between any of the samples, while the RP method showed moderate differences with partial overlap of the groups. These differences between methods are expected, since each method measures different mechanisms of antioxidant activity, solvent effects and assay conditions [64].

In the present study, the control kombucha samples showed a slightly higher in vitro antioxidant capacity than LAB–enriched samples when evaluated by DPPH and RP methods. These differences could be explained by interactions between LAB strains and the substrate, where certain polyphenolic compounds may be metabolised during fermentation. For example, Rodríguez et al. [65] reported that *L. plantarum* possesses the metabolic capacity to degrade specific phenolic compounds, which could explain why the *L. plantarum*–enriched sample exhibited the lowest total polyphenolic content. Similarly, Liang et al. [66] investigated fermentation of blue honeysuckle using *L. rhamnosus* and observed that total phenolic content initially increased but subsequently decreased, reaching levels lower than those at the beginning of fermentation after 24 h.

This reduction in polyphenolic content may also be attributed to interactions between polyphenolic compounds present in the matrix and macromolecular substances, such as whey proteins, which were used as carrier materials for LAB encapsulation in the enriched kombucha samples [66]. Polyphenolic compounds are known for their ability to form complexes with protein structures through hydrogen bonding and hydrophobic interactions, which can alter the overall detected antioxidant activity, as certain phenolic groups become less available to react in standard spectrophotometric assays [67]. De Morais et al. [68] demonstrated that complexation of whey protein isolate with epigallocatechin gallate resulted in suppressed reducing and antioxidant capacities.

Additionally, differences observed among LAB–enriched kombucha samples may be attributed to the use of different LAB strains, which possess distinct enzymatic profiles and therefore differ in their ability to metabolise phenolic compounds [65]. The obtained results agree with those reported by Nguyen et al. [25], who observed that three out of five free LAB–enriched kombucha samples exhibited lower DPPH radical scavenging activity compared to the control.

### 3.5. Analysis of Polyphenolic Compounds

The analysis of polyphenolic compounds showed that the addition of whey protein/LAB encapsulates affects the polyphenolic profile of kombucha. Syringic acid was the most dominant compound in all the samples (Table 6). The control sample had the highest concentration of gallic and caffeic acids, while all LAB–enriched samples showed a significant reduction of these compounds, which may be explained by LAB metabolic activities. For example, *L. plantarum* can decarboxylate gallic acid to pyrogallol [69], while Alberto et al. [70] noted that *L. hilgardii* consumes gallic acid, which stimulates their growth. On the other hand, syringic and ellagic acids, as well as flavonoids myricetin, quercetin and kaempferol, showed an increase in LAB–enriched samples compared to the control, with the most pronounced increase being noticed in myricetin and kaempferol. LAB can modify the flavonoid profile during fermentation, increasing the concentrations of aglycones such as myricetin and kaempferol in samples with LAB compared to controls [71]. This has also been shown in other plant fermentations where LAB increased the concentration of certain polyphenolic and flavonoid compounds, such as quercetin and kaempferol [72,73].

These results indicate that the addition of specific bacterial strains can modulate the polyphenolic profile of kombucha, with some compounds increasing significantly while others slightly decreasing, which may have implications for the nutritional and functional value of the beverage. Polyphenolic composition of kombucha generally varies across studies, which could be due to different geographical variations of the used substrates. Czarnowska-Kujawska et al. [33] also found the highest level of syringic acid in mint and nettle kombucha infusions and concluded that the fermentation positively affects the formation of syringic acid. Sun et al. [74] found that gallic and caffeic acids were the most dominant in traditionally prepared kombucha using black tea, as well as gallic and chlorogenic acids in a study by Ivanisova et al. [75]. It should be noted that the interpretations presented here are based on observed metabolite profiles and literature-supported pathways. Comprehensive metabolomics or pathway-level validation was not performed, and therefore, mechanistic conclusions should be considered indicative rather than definitive.

### 3.6. Antimicrobial Activity

Antimicrobial activity against tested microorganisms for all kombucha samples is presented in Table 7, Table 8, Table 9 and Table 10. Antimicrobial activity of the tested LAB–enriched kombucha samples showed a selective inhibitory effect, primarily against Gram-positive and certain Gram-negative bacteria, while inhibition of yeasts and moulds was absent in all samples. *L. plantarum*–enriched kombucha showed moderate antimicrobial activity, with more pronounced bacteriostatic activity, while bactericidal activity was recorded only for *Bacillus cereus*. A bit broader antibacterial spectrum was observed for the *L. hilgardii*–enriched sample, including inhibition of *Pseudomonas aeruginosa*. The most pronounced antibacterial activity was observed in the *L. rhamnosus*–enriched sample, where larger zones of inhibition against *Bacillus cereus*, *Salmonella* Typhimurium and *Staphylococcus aureus* were registered. The *L. rhamnosus*–enriched sample also had the highest TA [3] and *L. plantarum* the lowest, which could explain these differences. Antimicrobial activity in control samples was limited and present against *Staphylococcus aureus*, *Pseudomonas aeruginosa* and *Salmonella* Typhimurium, while inhibition of other tested microorganisms was not observed. Uninoculated media (black tea) did not show any antimicrobial activity.

Neutralised kombucha to pH 7 showed a complete or almost complete loss of antibacterial activity against most of the tested microorganisms. Exceptions were recorded for *Staphylococcus aureus* and *Salmonella* Typhimurium, where moderate inhibitory activity was maintained in some samples. Heat denaturation of kombucha led to a partial reduction of antimicrobial activity, whereby the inhibitory effect against bacterial test strains was recorded in most samples, while the activity against yeasts and moulds was still absent. Partial reduction of antimicrobial activity after heat treatment indicates that protein or peptide components, such as bacteriocins or bioactive peptides, may contribute to the overall inhibitory effect, but are not the only carriers of antimicrobial activity. By comparing untreated kombucha samples with the neutralised and heat-treated variants, it was observed that the highest antimicrobial activity was present in untreated samples, while NK and HTK showed a reduced effect, which indicates the synergistic action of acidity and thermostable components.

Antimicrobial activity of kombucha is mainly attributed to low pH values, especially due to the presence of acetic and other organic acids, as well as the presence of some proteins and bacteriocins that are present after fermentation of tea [49]. Molecules of organic acids can cause acidification of the cytoplasm and destruction of the bacterial cell. The antibacterial activity exhibited by the compounds in the kombucha drink can also be interpreted based on the osmotic pressure of the solutes that exist in the hypertonic environment in relation to the external aqueous environment. This facilitates the diffusion of bioactive substances from cell membranes through selective permeability. The lipophilic nature of some solutes facilitates their binding to bacterial cell membranes, which in turn causes cell death [76].

On the other hand, it is suggested that the antimicrobial activity of kombucha is not exclusively related to the presence of acetic acid, since the drink sometimes has an inhibitory effect at neutral pH on the growth of some microorganisms, including *Salmonella* typhimurium, as in the present study [1]. The results of the present study indicate that although acetic acid significantly affects the antibacterial activity of the samples, it is not the sole carrier of this activity. Better antimicrobial activity of LAB-encapsulated samples could be attributed to the presence of lactic acid in these samples. Lactic acid demonstrated antimicrobial activity against several Gram-negative and Gram-positive bacteria in previous studies, as well as yeasts, although in lower capacities [77]. *Lactobacillus* species can also produce low molecular mass antimicrobial metabolites and bacteriocins that could have also affected antibacterial activity in the present study [78].

The absence of an inhibitory effect against yeasts and moulds can be explained by their greater tolerance to an acidic environment and the presence of organic acids, as well as the fact that the LAB strains used probably do not produce antifungal metabolites in sufficient concentrations. The reduction in antimicrobial activity after pH neutralisation indicates a major contribution of organic acids, while the partial loss of activity following heat treatment suggests the involvement of heat-sensitive components, such as peptides. However, these observations provide indirect evidence only and do not allow quantitative differentiation of individual contributions. It must be emphasised that the agar well diffusion method provides a semi-quantitative assessment of antimicrobial activity; therefore, the results should be interpreted as indicative rather than definitive. Determination of minimal inhibitory concentration(s), as well as inclusion of standard antibiotic controls, would be required for precise quantification of antimicrobial potency and will be addressed in future studies. Similar results in terms of antibacterial activity are presented in a study by Battikh et al. [79] and Al-Mohammadi et al. [16], while antifungal activity was also noticed against *A. flavus* and *A. niger*, which is not in accordance with the present study.

### 3.7. Anti-Inflammatory Activity (AIA)

AIA in kombucha samples ranged between 49.33 ± 0.30% and 70.44 ± 0.61%, with *L. plantarum* and *L. hilgardii*–enriched samples exhibiting the lowest and *L. rhamnosus*–enriched samples the highest AIA (Table 11). Tukey’s HSD test revealed that the *L. rhamnosus*–enriched sample exhibited significantly higher AIA compared to all other samples (*p* < 0.05), while there was no significant difference between *L. plantarum* and *L. hilgardii*–enriched kombucha samples. The control sample showed moderate values, although significantly different from the enriched samples. Whey protein encapsulation did not increase AIA in a uniform pattern; rather, it seems that the effect was strain dependent.

Kombucha has previously demonstrated potential in modulating inflammatory responses, particularly through its influence on immune system regulation, as shown in previous studies, which could explain the still relatively high AIA [80]. For example, in a study conducted by Wang et al. [81], the anti-inflammatory activity of kombucha was studied in sepsis-induced mice. Orally administered kombucha has been shown to reduce levels of tumour necrosis factor-α (TNF-α) and interleukin (IL)-1b and IL-6, which are cytokines produced in response to inflammation. To the best of our knowledge, there is currently no available research addressing the effects of probiotic strain supplementation or encapsulation on the anti-inflammatory activity of kombucha. However, some studies have indicated that *L. rhamnosus* possesses strain-specific ability to produce bioactive metabolites with anti-inflammatory potential [82], which could explain the higher AIA results in the present study. *L. rhamnosus* and its metabolites can modulate immune cells, such as M1 macrophages and T lymphocytes, effectively suppressing the production of several pro-inflammatory cytokines, thereby regulating inflammatory responses and maintaining immune homeostasis through this dual mechanism of cytokine balance modulation [83]. The absence of significant differences between *L. plantarum* and *L. hilgardii* suggests a comparable impact of these strains on the inflammatory response under the applied fermentation conditions.

### 3.8. Antiproliferative Activity

**Figure 5:**
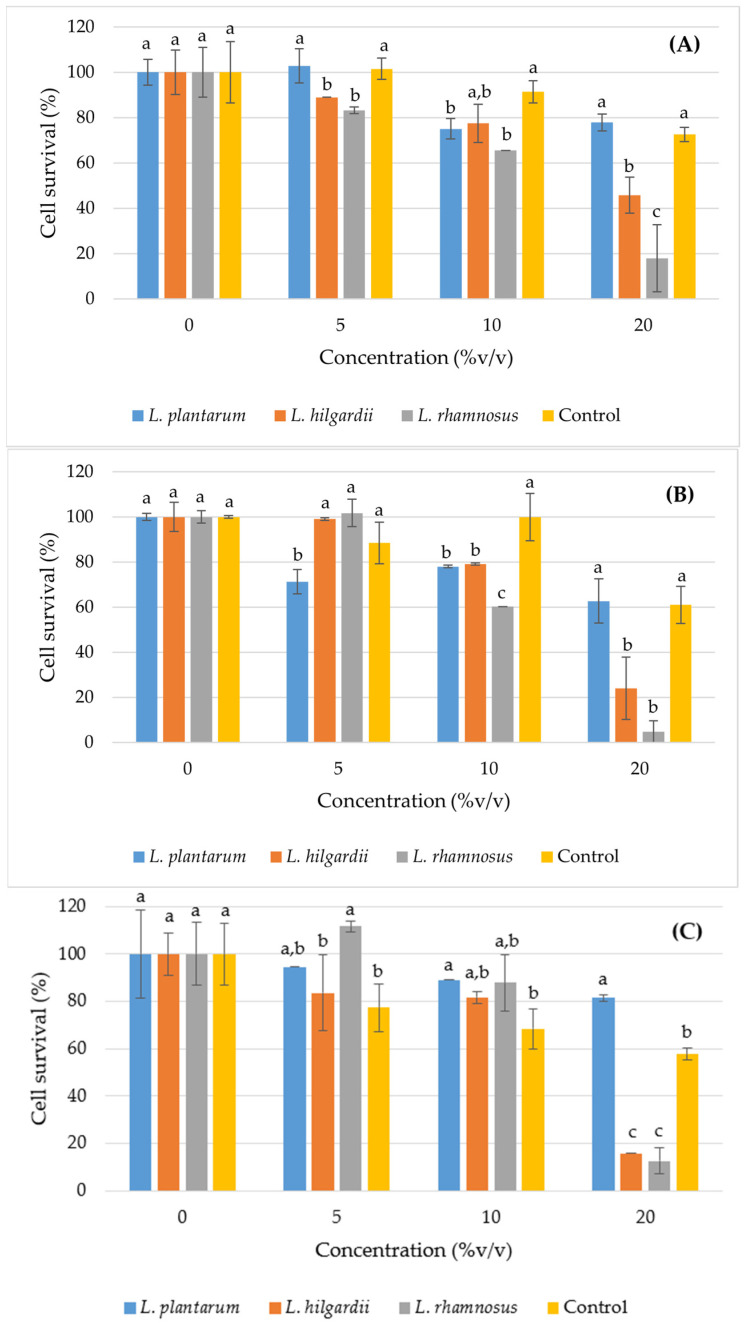
Antiproliferative activity against HeLa (**A**), HT-29 (**B**) and MCF-7 (**C**) cell lines. Values are expressed as mean ± SD (*n* = 3). Different letters indicate statistically significant differences among samples within the same concentration group according to Tukey’s HSD test (*p* < 0.05).

The antiproliferative activity of kombucha samples was evaluated using three tumour cell lines (HeLa, HT-29 and MCF-7) at increasing concentrations (5, 10 and 20% *v*/*v*). All results are expressed as a percentage of cell survival relative to the untreated control (0 = 100%) (Figure 5). The LAB–enriched samples showed a generally more pronounced antiproliferative activity compared to the control kombucha without the addition of encapsulated LAB cells.

A decrease in cell survival of HeLa cells was observed with increasing concentrations in all samples, indicating a dose response. The *L. rhamnosus*–enriched kombucha showed the most pronounced effect, with a decrease in survival to 17.886% at concentration 20% *v*/*v*, indicating a strong antiproliferative activity. The *L. hilgardii*–enriched sample also showed significant growth inhibition (45.73% at 20% *v*/*v*), while the *L. plantarum*–enriched sample showed a weaker effect (77.85% at 20% *v*/*v*). The control sample shows a weaker or inconsistent effect, with a higher percentage of survival compared to the *L. rhamnosus* and *L. hilgardii*–enriched samples. The HeLa cells appeared sensitive to treatment, especially to kombucha with encapsulated *L. rhamnosus*.

The HT-29 cells showed a pronounced sensitivity to several samples, especially at higher concentrations. The strongest antiproliferative effect was observed in the *L. rhamnosus*–enriched sample, with only 4.69% survival at a concentration 20% *v*/*v*. The *L. hilgardii*–enriched sample also showed a strong effect (24.02% at 20% *v*/*v*), while the *L. plantarum* had a moderate but clearly dose-dependent effect. The control kombucha sample retained a relatively high percentage of survival. The HT-29 cells were the most sensitive line in the present study, especially to samples with *L. rhamnosus* and *L. hilgardii*.

At a concentration of 20% *v*/*v*, a drastic drop in MCF-7 survival was observed for the *L. rhamnosus* and the *L. hilgardii*–enriched samples (12.63% and 15.76%, respectively). The *L. plantarum*–enriched sample showed weak activity with 81.54% survival at 20% *v*/*v*. The control kombucha sample caused a moderate decrease in MCF-7 cell survival. Overall, the *L. rhamnosus* sample showed the most pronounced and consistent antiproliferative activity on all three cell lines. The *L. hilgardii* had a strong but slightly weaker effect, while the *L. plantarum* showed weak antiproliferative potential, even less than the control kombucha sample.

The corresponding IC_50_ values, summarised in Table 12, quantitatively confirm these observations. *L. rhamnosus*–enriched sample exhibited the lowest IC_50_ values, indicating the highest antiproliferative potency among the tested samples, followed by *L. hilgardii*–enriched sample, while for *L. plantarum*–enriched sample and control, the IC_50_ was not reached within the tested concentration range (up to 20% *v*/*v*) and, therefore, they are not included in the table. It should be noted that these values reflect in vitro activity and do not provide mechanistic insight; further studies are needed to elucidate the underlying pathways, such as apoptosis or ROS induction.

Research hypothesised many possible mechanisms for the antiproliferative activity of kombucha. For example, the ability of this fermented beverage to act as an anti-cancer agent has been reported to be due to the presence of tea polyphenols and secondary metabolites produced during the fermentation process [49], which could explain the *L. rhamnosus*–enriched sample showing the highest antiproliferative effect, since this sample also had the highest concentration of phenols. Many studies have shown that the abilities of tea polyphenols present in this fermented beverage to inhibit gene mutations, inhibit the proliferation of cancer cells, induce apoptosis of cancer cells and have the ability to stop the formation of metastases [84]. In addition, several other compounds found in kombucha are believed to possess antiproliferative properties, such as glucuronic acid, gluconic acid, d-saccharic acid 1,4-lactone (DSL), acetic acid, lactic acid, ascorbic acid, succinic acid and vitexin [85]. Since the *L. rhamnosus*–enriched sample also showed the highest TA, the antiproliferative effects in the current study could also be due to the acidity of the samples. Other studies also confirmed the potential antiproliferative effects of kombucha beverages. For example, Cetojević-Simin et al. [86] examined the antiproliferative activity of *Satureja montana* L. tea kombucha, using the same cell lines as in the present study. It was concluded that the sample did not stimulate cell proliferation of the tested cell lines. In the HeLa cell line, the *Satureja montana* L. sample induced inhibition of cell growth by 20% at a lower concentration. In the research of Villarreal-Soto et al. [87], antiproliferative activity against MCF-7 breast cancer cell lines was also shown, as well as the fact that fermentation itself affects these activities. Caliskan et al. [88] investigated the antiproliferative effect of aronia, green tea and green tea-aronia kombucha samples, and the cell viability of HT-29 cells decreased by about 30%, 52% and 56%, respectively.

## 4. Conclusions

This study demonstrates that whey protein-based encapsulation of LAB can effectively maintain their viability during kombucha fermentation and modulate the chemical composition and bioactivity profile of the final product. LAB–enriched samples exhibited increased L-lactic acid production and enhanced in vitro antimicrobial, antiproliferative and anti-inflammatory activities in a strain-dependent manner. However, it is important to emphasise that these bioactivities were evaluated exclusively under in vitro conditions and therefore do not directly reflect physiological or clinical effects in humans. The observed functional properties should be interpreted as indicative of potential rather than confirmed health benefits. Overall, these findings support the feasibility of using encapsulated LAB as a strategy to develop kombucha with tailored bioactive profiles, while further in vivo and clinical investigations are necessary to substantiate their relevance for human health.

## Figures and Tables

**Table 1:** Organic acid contents of kombucha samples.

Kombucha Samples	Acetic Acid (mg/mL)	Malonic Acid (mg/mL)	Formic Acid (mg/mL)	Oxalic Acid (mg/mL)
*L. plantarum* + WP	1.1689 ± 0.0704 ^c^	0.2168 ± 0.0905 ^b,c^	0.1734 ± 0.0007 ^c^	0.2169 ± 0.0005 ^d^
*L. hilgardii* + WP	2.0734 ± 0.0042 ^a^	0.1202 ± 0.0020 ^c^	0.2112 ± 0.0025 ^b^	0.3198 ± 0.0002 ^c^
*L. rhamnosus* + WP	1.5646 ± 0.0664 ^b^	0.2997 ± 0.0097 ^b^	0.0742 ± 0.0045 ^d^	0.6011 ± 0.0006 ^a^
Control	2.1550 ± 0.0036 ^a^	0.4190 ± 0.0000 ^a^	0.2303 ± 0.0009 ^a^	0.4514 ± 0.0005 ^b^

Values are expressed as mean ± SD (*n* = 3). Different superscript letters within the same column indicate significant differences according to Tukey’s HSD test (*p* < 0.05).

**Table 2:** L, D-lactic acid contents of kombucha samples.

Kombucha Samples	L-Lactic Acid (g/L)	D-Lactic Acid (g/L)
*L. plantarum* + WP	0.90 ± 0.00 ^b^	0.98 ± 0.00 ^a^
*L. hilgardii* + WP	0.64 ± 0.01 ^c^	0.84 ± 0.01 ^b^
*L. rhamnosus* + WP	5.22 ± 0.09 ^a^	0.12 ± 0.01 ^c^
Control	0.02 ± 0.00 ^d^	0.02 ± 0.00 ^d^

Values are expressed as mean ± SD (*n* = 3). Different superscript letters within the same column indicate significant differences according to Tukey’s HSD test (*p* < 0.05).

**Table 3:** Vitamin C content in kombucha samples.

Kombucha Samples	Vitamin C (mg/L)
*L. plantarum* + WP	2.40 ± 0.16 ^b^
*L. hilgardii* + WP	2.24 ± 0.41 ^b^
*L. rhamnosus* + WP	2.92 ± 0.10 ^b^
Control	4.54 ± 0.98 ^a^

Values are expressed as mean ± SD (*n* = 3). Different superscript letters within the same column indicate significant differences according to Tukey’s HSD test (*p* < 0.05).

**Table 4:** Total polyphenol and flavonoid content of kombucha samples.

Kombucha Samples	Polyphenols (mg GAE/mL)	Flavonoids (mg RE/mL)
*L. plantarum* + WP	0.26 ± 0.00 ^c^	0.02 ± 0.00 ^a^
*L. hilgardii* + WP	0.29 ± 0.05 ^b,c^	0.01 ± 0.00 ^a^
*L. rhamnosus* + WP	0.39 ± 0.05 ^a^	0.01 ± 0.00 ^a^
Control	0.36 ± 0.01 ^a,b^	0.02 ± 0.00 ^a^

Values are expressed as mean ± SD (*n* = 3). Different superscript letters within the same column indicate significant differences according to Tukey’s HSD test (*p* < 0.05).

**Table 5:** Antioxidant activity of kombucha samples.

Kombucha Samples	DPPH (mmol TE/100 mL)	ABTS (mmol TE/100 mL)	RP (mmol TE/100 mL)
*L. plantarum* + WP	0.031 ± 0.004 ^c^	0.624 ± 0.118 ^a^	0.065 ± 0.003 ^b,c^
*L. hilgardii* + WP	0.079 ± 0.002 ^b^	0.689 ± 0.149 ^a^	0.071 ± 0.001 ^a,b^
*L. rhamnosus* + WP	0.081 ± 0.001 ^b^	0.692 ± 0.141 ^a^	0.074 ± 0.003 ^a^
Control	0.103 ± 0.001 ^a^	0.717 ± 0.114 ^a^	0.062 ± 0.005 ^c^

Values are expressed as mean ± SD (*n* = 3). Different superscript letters within the same column indicate significant differences according to Tukey’s HSD test (*p* < 0.05). DPPH—2,2-diphenyl-1-picrylhydrazyl, ABTS—2,2′-azino-bis-3-ethylbenzothiazoline-6-sulphonic acid, RP—reducing power (RP), TE—Trolox equivalents.

**Table 6:** Analysis of polyphenolic compounds in kombucha samples (µg/mL).

**Kombucha** **samples**	**Gallic acid**	**Syringic acid**	**Chlorogenic acid**	**Caffeic acid**	**Sinapic acid**
*L. plantarum* + WP	0.45 ± 0.00 ^d^	27.57 ± 0.00 ^a^	0.25 ± 0.00 ^b^	0.20 ± 0.00 ^b^	1.39 ± 0.00 ^a^
*L. hilgardii* + WP	1.83 ± 0.00 ^b^	25.94 ±0.00 ^c^	0.21 ±0.00 ^d^	0.18 ± 0.00 ^d^	0.11 ± 0.00 ^d^
*L. rhamnosus* + WP	0.85 ± 0.00 ^c^	26.32 ± 0.00 ^b^	0.23 ± 0.00 ^c^	0.20 ± 0.00 ^c^	0.97 ± 0.00 ^b^
Control	8.04 ± 0.00 ^a^	21.43 ± 0.00 ^d^	0.34 ± 0.00 ^a^	0.29 ± 0.00 ^a^	0.95 ± 0.00 ^c^
**Kombucha** **samples**	**Ellagic acid**	**Myricetin**	**Quercetin**	**Kaempferol**	
*L. plantarum* + WP	2.21 ± 0.00 ^a^	11.11 ± 0.00 ^a^	4.77 ± 0.00 ^c^	8.82 ± 0.00 ^a^	
*L. hilgardii* + WP	1.95 ± 0.00 ^c^	10.01 ± 0.00 ^c^	7.37 ± 0.00 ^b^	7.78 ± 0.00 ^c^	
*L. rhamnosus* + WP	2.10 ± 0.00 ^b^	10.53 ± 0.00 ^b^	7.86 ± 0.00 ^a^	8.45 ± 0.00 ^b^	
Control	1.63 ± 0.00 ^d^	5.95 ± 0.00 ^d^	4.44 ± 0.00 ^d^	3.76 ± 0.00 ^d^	

Values are expressed as mean ± SD (*n* = 3). Different superscript letters within the same column indicate significant differences according to Tukey’s HSD test (*p* < 0.05).

**Table 7:** Antibacterial activity of *L. plantarum*–enriched kombucha sample (mm).

**Test** **microorganism**	**Kombucha**		**Acetic acid (3.54 g/L)**	
	**A**	**B**	**A**	**B**
*B. cereus*	14.00 ± 0.00	nd	nd	nd
*S. aureus*	nd	16.00 ± 3.46	nd	16.00 ± 0.00
*L. monocytogenes*	nd	13.00 ± 0.00	nd	nd
*E. coli*	nd	14.33 ± 1.15	nd	18.00 ± 0.00
*S.* Typhimurium	nd	13.67 ± 0.58	nd	13.00 ± 0.00
*P. aeruginosa*	nd	12.67 ± 0.58	nd	nd
**Test** **microorganism**	**NK**		**HTK**	
	**A**	**B**	**A**	**B**
*B. cereus*	nd	nd	nd	nd
*S. aureus*	nd	20.33 ± 1.53	nd	18.00 ± 3.60
*L. monocytogenes*	nd	nd	nd	nd
*E. coli*	nd	nd	nd	14.67 ± 0.58
*S.* Typhimurium	nd	nd	nd	13.00 ± 0.00
*P. aeruginosa*	nd	nd	nd	nd

Values are expressed as mean ± SD (*n* = 3). A—bactericidal, B—bacteriostatic activity; NK—Neutralised kombucha; HTK—Heat treated kombucha; nd—not detected.

**Table 8:** Antibacterial activity of *L. hilgardii*–enriched kombucha sample (mm).

**Test** **microorganism**	**Kombucha**		**Acetic acid (4.83 g/L)**	
	**A**	**B**	**A**	**B**
*B. cereus*	14.00 ± 0.00	14.67 ± 0.58	12.00 ± 1.00	nd
*S. aureus*	nd	22.00 ± 2.00	nd	17.00 ± 0.00
*L. monocytogenes*	nd	nd	14.67 ± 0.58	nd
*E. coli*	nd	14.00 ± 1.00	nd	14.00 ± 0.00
*S.* Typhimurium	nd	14.67 ± 0.58	nd	13.68 ± 0.58
*P. aeruginosa*	13.68 ± 1.00	nd	13.00 ± 0.00	nd
**Test** **microorganism**	**NK**		**HTK**	
	**A**	**B**	**A**	**B**
*B. cereus*	nd	nd	nd	13.00 ± 0.00
*S. aureus*	nd	17.67 ± 0.58	nd	20.00 ± 1.00
*L. monocytogenes*	nd	nd	15.33 ± 0.58	nd
*E. coli*	nd	nd	nd	14.00 ± 1.00
*S.* Typhimurium	nd	nd	nd	14.00 ± 1.00
*P. aeruginosa*	nd	nd	13.00 ± 0.00	nd

Values are expressed as mean ± SD (*n* = 3). A—bactericidal, B—bacteriostatic activity; NK—Neutralised kombucha; HTK—Heat-treated kombucha; nd—not detected.

**Table 9:** Antibacterial activity of *L. rhamnosus*–enriched kombucha sample (mm).

**Test microorganism**	**Kombucha**		**Acetic acid (6.09 g/L)**	
	**A**	**B**	**A**	**B**
*B. cereus*	15.00 ± 0.00	19.00 ± 1.00	13.00 ± 1.00	nd
*S. aureus*	nd	23.33 ± 0.58	nd	15.00 ± 0.00
*L. monocytogenes*	16.00 ± 0.00	nd	16.00 ± 0.00	nd
*E. coli*	15.00 ± 1.00	nd	14.67 ± 0.58	nd
*S.* Typhimurium	14.67 ± 0.58	19.33 ± 2.08	15.33 ± 1.53	nd
*P. aeruginosa*	nd	12.00 ± 0.00	nd	12.00 ± 0.00
**Test microorganism**	**NK**		**HTK**	
	**A**	**B**	**A**	**B**
*B. cereus*	nd	nd	14.00 ± 1.00	nd
*S. aureus*	nd	20.67 ± 1.15	nd	21.67 ± 1.15
*L. monocytogenes*	nd	nd	13.00 ± 0.00	nd
*E. coli*	nd	nd	14.33 ± 1.15	
*S.* Typhimurium	nd	nd	nd	15.33 ± 0.58
*P. aeruginosa*	nd	nd	nd	nd

Values are expressed as mean ± SD (*n* = 3). A—bactericidal, B—bacteriostatic activity; NK—Neutralised kombucha; HTK—Heat treated kombucha; nd—not detected.

**Table 10:** Antibacterial activity of the control kombucha sample (mm).

**Test microorganism**	**Kombucha**		**Acetic acid (5.1 g/L)**	
	**A**	**B**	**A**	**B**
*B. cereus*	nd	nd	nd	nd
*S. aureus*	nd	20.00 ± 0.00	nd	nd
*L. monocytogenes*	nd	nd	nd	nd
*E. coli*	nd	nd	nd	nd
*S.* Typhimurium	nd	17.33 ± 0.58	nd	11.00 ± 0.00
*P. aeruginosa*	13.00 ± 0.00	nd	13.00 ± 0.00	nd
**Test microorganism**	**NK**		**HTK**	
	**A**	**B**	**A**	**B**
*B. cereus*	nd	nd	nd	nd
*S. aureus*	nd	20.00 ± 0.00	nd	20.00 ± 0.00
*L. monocytogenes*	nd	nd	nd	nd
*E. coli*	nd	nd	nd	nd
*S.* Typhimurium	nd	17.33 ± 1.53	nd	17.33 ± 1.53
*P. aeruginosa*	nd	nd	nd	nd

Values are expressed as mean ± SD (*n* = 3). A—bactericidal, B—bacteriostatic activity; NK—Neutralised kombucha; HTK—Heat-treated kombucha; nd—not detected.

**Table 11:** Anti-inflammatory activity of kombucha samples.

**Kombucha Samples**	**AIA (%)**
*L. plantarum* + WP	49.33 ± 0.30 ^c^
*L. hilgardii* + WP	49.53 ± 1.66 ^c^
*L. rhamnosus* + WP	70.44 ± 0.61 ^a^
Control	63.52 ± 0.40 ^b^

Values are expressed as mean ± SD (*n* = 3). Different superscript letters within the same column indicate significant differences according to Tukey’s HSD test (*p* < 0.05).

**Table 12:** Results of the MTT assay in terms of IC_50_ values (% *v*/*v*) after 48 h of continuous action in human cancer cells.

Kombucha Samples	IC_50_ (% *v*/*v*)		
	HeLa	HT-29	MCF-7
*L. hilgardii* + WP	18.65 ± 0.60	15.29 ± 0.56	14.93 ± 2.47
*L. rhamnosus* + WP	13.31 ± 1.43	11.74 ± 1.07	14.40 ± 2.26

Values are expressed as mean ± SD (*n* = 3). For *L. plantarum* + WP and Control, the IC_50_ was not reached within the tested concentration range (up to 20% *v*/*v*), and therefore, they are not included in the table.

## Data Availability

The original contributions presented in the study are included in the article. Further inquiries can be directed to the corresponding author.
